# Does poor dental health predict becoming homebound among older Japanese?

**DOI:** 10.1186/s12903-016-0209-9

**Published:** 2016-04-30

**Authors:** Shihoko Koyama, Jun Aida, Katsunori Kondo, Tatsuo Yamamoto, Masashige Saito, Rika Ohtsuka, Miyo Nakade, Ken Osaka

**Affiliations:** Department of International and Community Oral Health, Tohoku University Graduate School of Dentistry, Sendai City, Miyagi Japan; Center for Preventive Medical Sciences, Chiba University, Chiba City, Chiba Japan; Department of Gerontological Evaluation, Center for Gerontology and Social Science, National Center for Geriatrics and Gerontology, Obu City, Aichi Japan; Division of Dental Sociology, Department of Oral Science, Graduate School of Dentistry, Kanagawa Dental University, Yokosuka City, Kanagawa Japan; Department of Social Welfare, Nihon Fukushi University, Nagoya City, Aichi Japan; Doctoral Institute for Evidence Based Policy, Tokyo, Japan; Department of Health and Nutrition, Faculty of Health and Nutrition, Tokaigakuen University, Nagoya City, Aichi Japan

**Keywords:** Community dentistry, Dental public health, Epidemiology, Homebound

## Abstract

**Background:**

Being homebound is an important risk factor of functional disability in older people. There is a possibility of bidirectional relationship between homeboundness and dental health. This prospective cohort study examined the association of dental health, which includes social function, on homeboundness in the future.

**Methods:**

The participants were ≥ 65 years, responded to two postal surveys conducted in 2006 and 2010, and were not homebound at baseline. Logistic regression analysis was used to estimate the odds ratios for homeboundness, defined as going out of one’s home less than once weekly. Self-reported baseline dental status was used as the main predictor. Age, sex, marital status, educational attainment, income, comorbidity, depression, walking time, living alone, and area of residence were used as covariates.

**Results:**

Among 4390 non-homebound respondents, 7.4 % were homebound four years later. The proportions of homebound respondents with < 20 teeth without dentures, < 20 teeth with dentures, and ≥ 20 teeth were 9.7, 8.8, and 4.4 %, respectively. The odds for being homebound in the 65–74-year age group, adjusted for covariates, was 1.78 (95 % CI: 1.01–3.13; *p* < 0.05) times higher for respondents with < 20 teeth and no dentures than that for respondents with ≥ 20 teeth. Among the participants in the ≥ 75-year age group, a significant association of homeboundness and dental health was not observed.

**Conclusions:**

Among the young-old population, poor dental health predicted future onset of homeboundness, while depressive symptoms did not show any significant association.

## Background

Being homebound leads to social isolation and physical inactivity in older people [1], which are important risk factors of functional disability, defined as a difficulty in performing activities of daily living. Functional disability has attracted increased attention as a public health problem in many aging societies [1]. In addition, previous studies have shown that homebound older people are at a significantly higher risk of mortality than non-homebound older people [1–3]. The various sociodemographic and behavioral risk factors of homeboundness include older age [2, 4–6], male sex [2], lower socioeconomic status [4, 6], unmarried status (single, separated, or divorced) [7], living alone [2, 4, 8], and poorer physical and psychological health [2, 4, 7].

A previous study reported the possibility that homebound people had poor dental health because of lack of access to dental care. In a study of homebound older adults living in New York City, the participants had poor dental health, most of whom did not have access to dental care [9]. In addition to the possibility that homeboundness causes poor dental health, there is a possibility that poor dental health causes homeboundness thorough several pathways. Dental health may affect both the physical and social behaviors of homebound individuals. Dental health affects not only physical health status but also social abilities. Dental health plays an important role in food choice and nutritional intake [10–15]. Additionally, recent studies have shown the effects of dental health on general health status include increased incidence of falls [16, 17] and functional disability [18]. In addition to these physical health factors, dental health, including loss of teeth, also affects social factors such as conversation [19] and facial attractiveness [20]. Embarrassment is often experienced when people have poor dental health issues such as having fewer remaining teeth [21]. Therefore, poor dental health could reduce social participation [16]. As a result of these mechanisms, inadequate dental health may have a negative effect on social activities, leading individuals to isolate themselves from others. Although there is a possibility of a bidirectional relationships between homeboundness and dental health, homeboundness causes poor dental health and poor dental health causes homeboundness, no study has examined the later direction, association of dental health on homeboundness.

If homeboundness and dental health are bidirectionally related, the negative spiral of homeboundness and poor dental health could deteriorate the activity of older people. In this situation, maintaining good dental health could alleviative this negative spiral. The aim of this longitudinal cohort study was to examine the association between poor dental health (tooth loss and lack of dentures) and being homebound in older Japanese people. We hypothesized that poor dental health at baseline predicts the homebound status at follow-up.

## Methods

### Respondents and setting

This prospective cohort study used data from the Japan Gerontological Evaluation Study (JAGES) Project [22, 23]. The JAGES Project investigated social, behavioral, and health factors in people aged 65 years or older. The JAGES sample was restricted to people who did not already have physical or cognitive disabilities, which were defined as those without eligibility for receiving long-term public care insurance benefits. Thus, the goal of the JAGES Project was to assess the health status and social determinants of able-bodied people aged 65 years or older. The present longitudinal study used panel data from surveys conducted in 2006 and 2010.

**Fig. 1:**
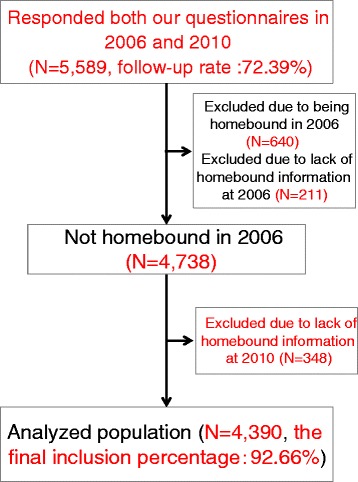
Data for 4390 respondents were included in the analysis

We mailed self-reported questionnaires to people who were randomly selected from the list of older residents not receiving long-term public care insurance benefits in each municipality. However, as the study fields were not randomly selected from whole Japanese municipalities, we did not apply weight analysis. In 3 municipalities where the dental health questionnaires were distributed for both study periods, 7270 individuals completed the questionnaire in 2006, and 5589 responded to our questionnaire in 2010 (follow-up rate: 72.39 %). The present study determined which respondents had become homebound between 2006 and 2010, and we excluded 640 individuals who were homebound at baseline (2006). Of these respondents, 211 were excluded because of a lack of information regarding being homebound in 2006, 348 were excluded because of a lack of information regarding being homebound in 2010. Finally, 92.66 % of the participants were included in our analyses. Data collected from 4390 respondents were included in the analysis (Fig. 1).

### Ethical considerations

The JAGES protocol was reviewed and approved by the Ethics Committee on Research of Human Subjects of Nihon Fukushi University. The questionnaire was sent via mail, with a written explanation of the study aim. The people who returned the questionnaire were regarded as having provided consent to participate in the survey. The authors obtained the permission to use the data from the JAGES data management committee.

### Outcome variables

Homeboundness has several similar definitions. The criteria of homeboundness include going out of one’s home once weekly or less often [4], less often than once weekly [24] and once monthly or less often [25]. Since being homebound was considered to be affected by social and cultural background, we applied the definition used in Japan, which is going out of one’s home less often than once weekly [24]. This reflects not only physical reasons for being confined to one’s home, such as being bedridden, but also psychosocial or geographical reasons. Frequency of going outdoors was measured by using the question, “How often do you usually go outside of the house (this includes shopping, meeting with people, walking, going to the hospital, and other activities)?”

### Explanatory variables

The number of remaining teeth and the use of dentures at baseline (2006) were used as variables representing dental health status. They were assessed by responses to the self-administered questionnaire. Previous studies have indicated that older people with 20 or more teeth ingested more food relative to those with fewer than 20 teeth [26]. Therefore, we categorized the numbers of remaining teeth as follows: 20 or more teeth, fewer than 20 teeth with dentures, and fewer than 20 teeth without dentures.

### Covariates

From previous studies, we selected physical, psychological, and geographical variables as the covariates [1, 7, 27, 28]. As in the previous studies, the following questions regarding sociodemographic characteristics, baseline health status, and risk factors of homeboundness were included in the analyses as covariates: age, sex, marital status, educational attainment, annual household income, comorbidity, depression, walking time (min/day), living alone, and area of residence at baseline (2006). To minimize the effect of age as a strong confounding factor, we conducted a stratified analysis according to age and included age as a continuous variable in the models. Marital status was categorized as married, widowed, separated, never married, and other. Educational attainment was categorized as follows: < 6 years, 6–9 years, 10–12 years, and ≥ 13 years. Annual household income was categorized as follows: < $20,000 (< ¥2,000,000), $20,000–$29,999 (¥2,000,000–¥2,999,999), $30,000–$39,999 (¥3,000,000–¥3,999,999), and ≥ $40,000 (≥¥4,000,000) (US$1 = ¥100). Comorbidity was measured by using the question, “Do you receive treatment now?” (to which respondents answered yes or no). Depressive symptoms were measured via the short version of the Geriatric Depression Scale (GDS-15) by using a simple yes/no format suitable for self-administration. Previous studies have concluded that a cutoff point of 5 is appropriate for use with the GDS as a screening tool for depression in community-dwelling older adults in the United States and Japan [29, 30]. Therefore, a cutoff point of 5 was used as an indication of depression in this study. Walking time was categorized as follows: < 30, 30–59, 60–89, and ≥ 90 min/day. The categories representing the numbers of people living in respondents’ households were “living alone” or “living with others.” Because the risk of being homebound was affected by residential area, residential municipality was also used as a categorical covariate as follows: living area A, living area B, or living area C. The 2005 population densities [31] for areas A, B, and C were 1,026.6, 566.9, and 572.9 persons/km^2^, respectively.

### Statistical analysis

We calculated prevalence and 95 % confidence interval (CI) for respondents who were homebound due to dental health. We then calculated the odds ratios (ORs) of the baseline dental health status for homeboundness four years later. Univariate and multivariate ORs and 95 % CIs for dental health were calculated. As age was strongly associated with both dental health and homebound status, we applied a stratified analysis according to age. The participants were stratified into two groups as follows: 65–74 years old or 75 years or older. In the multivariate model, age, sex, marital status, educational attainment, annual household income, comorbidity, depression (GDS-15), walking time (min/day), living alone, and area of residence at baseline were included. We put emphasis on the theoretical importance of the covariates, and included all covariates in the multivariate model. If data were missing for explanatory variables, the corresponding observations were assigned to “missing” categories. In the present analysis, we selected the study population from three municipalities. The sampling rates and populations of the municipalities were similar. As a result, the sampling weight was not applied in the analyses. All analyses were performed by using SPSS version 22.0 statistical software.

In addition, the results of the logistic regression were used to calculate the population-attributable fraction (PAF) of dental health for being homebound. The PAF is defined as “the proportional reduction in population disease or mortality that would occur if exposure to a risk factor were reduced to an alternative ideal exposure scenario (e.g., no tobacco use)” by the World Health Organization [32]. The PAF has been used to examine the impact of risk factors on health outcomes in various public health studies [33, 34]. The impact of dental health on the population’s risk of being homebound was estimated according to the PAF concept.

## Results

Of 4390 respondents, whose mean (SD) age was 72.37 years (5.44), 2035 men and 2355 women were not homebound at baseline, 7.4 % (*n* = 324) of whom were homebound four years after baseline assessment. The baseline characteristics of the respondents according to homebound status at follow-up are presented in Table 1. The proportion of homebound respondents with fewer than 20 teeth and no dentures, fewer than 20 teeth and dentures, and 20 or more teeth were 9.7, 8.8, and 4.4 %, respectively. Respondents who were older, lowest educational attainment, shorter walking time (min/day), had lived alone, exhibited symptoms of depression, had received hospital treatment, earned the lowest annual household incomes, or lived in areas B or C were more likely to be homebound.

**Table 1:** Baseline characteristics of the participants according to homebound status at follow-up (*n* = 4390)

	Number of respondents (%) (excluding those who were homebound at baseline)					
	65–74 years old (*n* = 3007)			≥75 years old (*n* = 1383)		
	Total	Non-homebound	Homebound	Total	Non-homebound	Homebound
Sex						
Male	1410 (100.0)	1338 (94.9)	72 (5.1)	625 (100.0)	550 (88.0)	75 (12.0)
Female	1597 (100.0)	1530 (95.8)	67 (4.2)	758 (100.0)	648 (85.5)	110 (14.5)
Dental health						
≥20 teeth	1195 (100.0)	1158 (96.9)	37 (3.1)	298 (100.0)	270 (90.6)	28 (9.4)
≤19 teeth with dentures	1465 (100.0)	1388 (94.7)	77 (5.3)	922 (100.0)	789 (85.6)	133 (14.4)
≤19 teeth without dentures	297 (100.0)	275 (92.6)	22 (7.4)	127 (100.0)	108 (85.0)	19 (15.0)
Marital status						
Marriage	2424 (100.0)	2318 (95.6)	106 (4.4)	810 (100.0)	700 (86.4)	110 (13.6)
Widowed	410 (100.0)	388 (94.6)	22 (5.4)	475 (100.0)	417 (87.8)	58 (12.2)
Separated	44 (100.0)	42 (95.5)	2 (4.5)	9 (100.0)	9 (100.0)	0 (0.0)
Never married	33 (100.0)	31 (93.9)	2 (6.1)	18 (100.0)	13 (72.2)	5 (27.8)
Other or missing	96 (100.0)	89 (92.7)	7 (7.3)	71 (100.0)	59 (83.1)	12 (16.9)
Education						
<6 years	19 (100.0)	18 (94.7)	1 (5.3)	61 (100.0)	52 (85.2)	9 (14.8)
6–9 years	1563 (100.0)	1473 (94.2)	90 (5.8)	708 (100.0)	600 (84.7)	108 (15.3)
10–12years	892 (100.0)	860 (96.4)	32 (3.6)	375 (100.0)	337 (89.9)	38 (10.1)
≥13 years	377 (100.0)	366 (97.1)	11 (2.9)	133 (100.0)	116 (87.2)	17 (12.8)
Annual household income^a^						
<$20,000	1190 (100.0)	1123 (94.4)	67 (5.6)	496 (100.0)	426 (85.9)	70 (14.1)
$20,000–29,999	662 (100.0)	639 (96.5)	23 (3.5)	246 (100.0)	215 (87.4)	31 (12.6)
$30,000–39,999	471 (100.0)	456 (96.8)	15 (3.2)	161 (100.0)	142 (88.2)	19 (11.8)
≥$40,000	301 (100.0)	288 (95.7)	13 (4.3)	133 (100.0)	117 (88.0)	16 (12.0)
Do you have hospital treatment?						
Yes	1933 (100.0)	1846 (95.5)	87 (4.5)	961 (100.0)	820 (85.3)	141 (14.7)
No	794 (100.0)	762 (96.0)	32 (4.0)	211 (100.0)	186 (88.2)	25 (11.8)
Depression (GDS score)						
0–4	1961 (100.0)	1878 (95.8)	83 (4.2)	811 (100.0)	718 (88.5)	93 (11.5)
≥5	575 (100.0)	536 (93.2)	39 (6.8)	246 (100.0)	203 (82.5)	43 (17.5)
Walking time (min/day)						
<30	840 (100.0)	788 (93.8)	52 (6.2)	433 (100.0)	358 (82.7)	75 (17.3)
30–59	959 (100.0)	917 (95.6)	42 (4.4)	478 (100.0)	420 (87.9)	58 (12.1)
60–89	460 (100.0)	444 (96.5)	16 (3.5)	203 (100.0)	185 (91.1)	18 (8.9)
≥90	641 (100.0)	616 (96.1)	25 (3.9)	201 (100.0)	178 (88.6)	23 (11.4)
Do you stay with your family?						
Yes	2477 (100.0)	2365 (95.5)	112 (4.5)	1023 (100.0)	894 (87.4)	129 (12.6)
No (living alone)	185 (100.0)	173 (93.5)	12 (6.5)	127 (100.0)	111 (87.4)	16 (12.6)
Living area^b^						
A	1277 (100.0)	1251 (98.0)	26 (2.0)	488 (100.0)	449 (92.0)	39 (8.0)
B	923 (100.0)	873 (94.6)	50 (5.4)	429 (100.0)	359 (83.7)	70 (16.3)
C	807 (100.0)	744 (92.2)	63 (7.8)	466 (100.0)	390 (83.7)	76 (16.3)

^a^US $1 = \100

^b^The population densities of areas A, B, and C were 1,026.6, 566.9, and 572.9 persons/km^2^ respectively

*GDS* Geriatric Depression Scale

Table 2 shows the results of the age-stratified logistic regression analyses. In the univariate analysis, the odds of being homebound for respondents with fewer than 20 teeth and no dentures in the 65- to 74-year age group was 2.50 (95 % CI: 1.45–4.31) times greater than that for respondents with 20 or more teeth. Even after adjustment for a number of covariates, the odds of being homebound for respondents with fewer than 20 teeth and no dentures in the 65- to 74-year age group was 1.78 (95 % CI: 1.01–3.13) times greater than that for respondents with 20 or more teeth. In contrast, among the participants aged 75 years or older, no significant association was observed between dental health and homeboundness. Widowed participants had lower odds for homeboundness in the 75-year or older age group. Although statistically non-significant, depressive participants tended to being homebound for both age groups. The PAF for dental health of the respondents with fewer than 20 teeth in the 65- to 74-year age group was 18.72 %. This was equivalent to avoidance of being homebound by preventing tooth loss in 187 per 1000 cases.

**Table 2:** Association between dental health at baseline and homeboundness at follow-up (*n* = 4390)

	65–74 years old (*n* = 3007)		≥75 years old (*n* = 1383)	
	Univariate analysis	Multivariate analysis^a^	Univariate analysis	Multivariate analysis^a^
Age: continuous	1.12 (1.05–1.19)***	1.09(1.02–1.16)*	1.10 (1.05–1.14)***	1.10(1.05–1.15)***
Sex				
Male	1.00 (reference)	1.00 (reference)	1.00 (reference)	1.00 (reference)
Female	0.81 (0.58–1.14)	0.68(0.47–0.99)*	1.24 (0.91–1.71)	1.40(0.96–2.03)
Dental health				
≥ 20 teeth	1.00 (reference)	1.00 (reference)	1.00 (reference)	1.00 (reference)
≤ 19 teeth with dentures	1.74 (1.16–2.59)**	1.39(0.92–2.10)	1.63 (1.06–2.50)*	1.40(0.89–2.21)
≤ 19 teeth without dentures	2.50 (1.45–4.31)***	1.78(1.01–3.13)*	1.70 (0.91–3.17)	1.47(0.76–2.84)
Marital status				
Marriage	1.00 (reference)	1.00 (reference)	1.00 (reference)	1.00 (reference)
Widowed	1.24 (0.77–1.99)	1.21(0.67–2.19)	0.89 (0.63–1.24)	0.56(0.37–0.86)**
Separated	1.04 (0.25–4.36)	1.08(0.24–4.79)	-	-
Never married	1.41 (0.33–5.97)	1.09(0.23–5.16)	2.45 (0.86–7.00)	2.27(0.68–7.53)
Other or missing	1.24 (0.77–1.99)	1.26(0.54–2.94)	0.89 (0.63–1.24)	0.83(0.40–1.72)
Education (years)				
< 6	1.85 (0.23–15.11)	0.96(0.11–8.55)	1.18 (0.49–2.82)	0.80(0.31–2.06)
6–9	2.03 (1.08–3.84)*	1.47(0.76–2.86)	1.23 (0.71–2.13)	1.06(0.59–1.91)
10–12	1.24 (0.62–2.48)	1.18(0.58–2.40)	0.77 (0.42–1.42)	0.70(0.36–1.33)
≥13	1.00 (reference)	1.00 (reference)	1.00 (reference)	1.00 (reference)
Annual household income^b^				
< $20,000	1.32 (0.72–2.43)	0.93 (0.49–1.76)	1.20 (0.67–2.15)	0.89 (0.48–1.66)
$20,000–29,999	0.80 (0.40–1.60)	0.73 (0.36–1.49)	1.05 (0.55–2.01)	0.82 (0.42–1.62)
$30,000–39,999	0.73 (0.34–1.55)	0.70 (0.32–1.50)	0.98 (0.48–1.99)	0.91 (0.44–1.91)
$40,000 ≤	1.00 (reference)	1.00 (reference)	1.00 (reference)	1.00 (reference)
Do you have hospital treatment?				
Yes	1.12 (0.74–1.70)	1.88(0.73–4.82)	1.28 (0.81–2.01)	1.26(0.78–2.03)
No	1.00 (reference)	1.00 (reference)	1.00 (reference)	1.00 (reference)
Depression (GDS score)				
0–4	1.00 (reference)	1.00 (reference)	1.00 (reference)	1.00 (reference)
≥5	1.65 (1.11–2.44)*	1.44(0.95–2.18)	1.64 (1.10–2.42)*	1.39(0.92–2.12)
Walking time (min/day)				
< 30	1.63 (1.00–2.65)	1.46(0.88–2.42)	1.62 (0.98–2.67)	1.56(0.93–2.64)
30–59	1.13 (0.68–1.87)	1.08(0.64–1.82)	1.07 (0.64–1.79)	1.11(0.65–1.90)
60–89	0.89 (0.47–1.68)	0.90(0.47–1.73)	0.75 (0.39–1.44)	0.82(0.42–1.61)
≥90	1.00 (reference)	1.00 (reference)	1.00 (reference)	1.00 (reference)
Do you stay with your family?				
Yes	1.00 (reference)	1.00 (reference)	1.00 (reference)	1.00 (reference)
No (living alone)	1.46 (0.79–2.71)	1.16(0.53–2.53)	1.00 (0.57–1.74)	1.10(0.57–2.10)
Living area^c^				
A	1.00 (reference)	1.00 (reference)	1.00 (reference)	1.00 (reference)
B	2.76 (1.70–4.46)***	2.64(1.62–4.29)***	2.24 (1.48–3.40)***	2.21(1.44–3.40)***
C	4.07 (2.56–6.49)***	3.55(2.20–5.74)***	2.24 (1.49–3.38)***	2.10(1.38–3.21)***

^a^Adjusted for age, sex, marital status, educational attainment, annual household income, comorbidity, depression (Geriatric Depression Scale score), walking time (min/day), living alone, and living area at baseline (2006)

^b^US $1 = \100

^c^The population densities of areas A, B, and C were 1,026.6, 566.9, and 572.9 persons/km^2^, respectively.

Data are presented as odds ratios (95 % confidence intervals), p value of homeboundness of the respondents

*GDS* Geriatric Depression Scale

*: *p* < 0.05, **: *p* < 0.01, ***: *p* < 0.005

## Discussion

To the best of our knowledge, this is the first study to examine the association between dental health at baseline and homebound status at follow-up in older people. The result of a longitudinal data analysis suggested that, in the 65- to 74-year age group, having fewer teeth and no dentures at baseline was associated with future risk of being homebound. In contrast, in the 75-year or older age group, no significant association was observed between dental health and homeboundness. Therefore, for young-old people, dental health was an important predictor of being homebound. Future intervention studies focused on improving dental health in order to prevent homeboundness in older Japanese population are required.

Being homebound also showed robust associations with living area in both age groups. Previous studies in Japan reported that higher population density, a proxy of possibility for social interactions, was associated with lower prevalence of homeboundness [28]. An intervention that aims to increase social interactions would be beneficial in areas with higher prevalence of homeboundness [35]. Even after adjustments for living area, the association of dental health and homeboundness was still significant among young-old people. Compared to living area, dental health is a relatively easy target for modification in public health programs.

Based on the results of previous studies and the present study, both pathway directions could exist between homeboundness and dental health. Older people who were homebound and/or had functional disabilities had limited access to dental care [9]. Therefore, homebound people tend to have poor dental health [8]. In addition, the present study suggests that poor dental health increases the risk of homeboundness. Several possible pathways may link dental health and homeboundness. Dental health, including loss of teeth, also affects food choice and nutritional intake [10–15], conversation [19], and facial attractiveness [20]. Therefore, poor dental health could reduce social participation [16]. A cross-sectional study conducted in 2013 demonstrated the association between social participation and dental health [36]. Another cross-sectional study reported that denture use was significantly associated with participation in social groups among community-dwelling older Japanese women after adjusting for possible confounders [37]. Through these mechanisms, inadequate dental health may have a negative influence on social activities, leading individuals to isolate themselves from others. From a physical perspective, poor dental health can lead to general health problems, including functional disability and dementia [38]. Loss of physical or psychological health is associated with the risk of becoming homebound [2, 4, 7, 8].

In this study, the association of poor dental health and homebound status was stronger in the young-old population. This result may be explained by social compassion related to dental health. Among participants, the percentages of people with 20 or more teeth among 74 or younger and 75 or older were 40 and 22 %, respectively. A smaller number of people had poor dental health within the young-old population. Therefore, poor dental health status should be more severe in the young-old population. A psychological study reported that in a given situation, performance negatively judged by others initiated the highest psychological stress responses [39]. Among the young-old population, poor dental health reflects more negatively on facial attractiveness compared to the older population. This situation could erode self-esteem, decreasing social interaction and increasing homeboundness.

The implications of this study are that interventions that promote dental health and denture use may prevent older people from becoming homebound. In this study, 9.7 % of the respondents did not use dentures in spite of having fewer than 20 teeth. Improving the rate of denture use among older people with fewer teeth could reduce their risk of homeboundness in the future. Socioeconomic inequalities were observed in dental prosthesis use among older Japanese people [40]. In addition to dental health, homeboundness is associated with socioeconomic conditions [1]. Therefore, up-stream public health approaches that consider a broad range of social determinants of health are needed to prevent homeboundness and deterioration of dental health [41, 42].

The strength of our study includes a prospective cohort design involving the use panel data. This design was suitable for the inference of causality. In addition, our analysis considered various covariates that previous studies found to be associated with homeboundness. Even after adjustments for these covariates, a significant association was found between dental health and homeboundness.

Our findings should be considered within the context of its limitations. First, the follow-up rate was relatively low (72.39 %). Although follow-up rates of 50–80 % have been shown to be acceptable [43, 44], higher rates are desirable. Since our study respondents were 65 years or older, their physical conditions were sometimes unstable. In fact, the characteristics of non-respondent to the follow-up survey were younger and healthier (Table 3). Therefore, lower follow-up rates decrease the generalizability of the present results. Second, dental health (number of remaining teeth and denture use) was self-reported, and even though the validity of this measure has been well established with respect to objective measures [45–48], self-reported dental health was found to be imprecise relative to clinical dental checkups. Therefore, our result regarding the association between dental health and homebound status is considered an underestimation. Third, the study fields were not randomly selected from whole Japanese municipalities; thus, we did not apply weight analysis. Our participants did not include people with disability at baseline. Therefore, the generalizability of the present results to the Japanese population is limited. Fourth, we did not examine dental attendance history at the baseline survey. Including this variable as a covariate can further reduce the possibility of reverse causation.

**Table 3:** Baseline characteristics of the respondents and non-respondents to the follow-up survey (*n* = 7270)

	Number (%) at baseline	
	Respondents to the follow-up (*n* = 5589)	Non-respondents to the follow-up(*n* = 1681)
Sex		
Male	2513(45.0)	785(46.7)
Female	3076(55.0)	896(53.3)
Dental health		
≥20 teeth	1788(32.0)	405(24.1)
≤19 teeth with dentures	3064(54.8)	927(55.1)
≤19 teeth without dentures	579(10.4)	240(14.3)
Marital status		
Marriage	4020(71.9)	1147(68.2)
Widowed	1140(20.4)	330(19.6)
Separated	68(1.2)	18(1.1)
Never married	67(1.2)	28(1.7)
Other or missing	294(5.3)	158(9.4)
Education		
<6 years	129(2.3)	52(3.1)
6–9 years	2976(53.2)	995(59.2)
10–12years	1517(27.1)	325(19.3)
≥13 years	587(10.5)	122(7.3)
Annual household income^a^		
<$20,000	2198(39.3)	743(44.2)
$20,000–29,999	1065(19.1)	213(12.7)
$30,000–39,999	741(13.3)	152(9.0)
≥$40,000	511(9.1)	125(7.4)
Do you have hospital treatment?		
Yes	3685(65.9)	1014(60.3)
No	1230(22.0)	409(24.3)
Depression (GDS score)		
0–4	3311(59.2)	823(49.0)
≥5	1107(19.8)	351(20.9)
Walking time (min/day)		
<30	1705(30.5)	549(32.7)
30–59	1738(31.1)	453(26.9)
60–89	805(14.4)	221(13.1)
≥90	1019(18.2)	281(16.7)
Do you stay with your family?		
Yes	394(7.0)	102(6.1)
No (living alone)	4339(77.6)	1220(72.6)
Living area^b^		
A	2118(37.9)	410(24.4)
B	1661(29.7)	515(30.6)
C	1810(32.4)	756(45.0)

^a^US $1 = \100

^b^The population densities of areas A, B, and C were 1,026.6, 566.9, and 572.9 persons/km^2^ respectively

*GDS* Geriatric Depression Scale

## Conclusions

This prospective cohort study demonstrated that having fewer teeth and failure to use dentures was associated with future onset homeboundness in young-old population. Among this young-old population, the association between dental health and homeboundness was stronger than association with depressive symptoms.

### Availability of supporting data

Data are from the JAGES study. All enquiries are to be addressed at the data management committee via e-mail: dataadmin@jages.net. All JAGES datasets have ethical or legal restrictions for public deposition due to inclusion of sensitive information from the human participants.

## Abbreviations

CI: confidence interval
GDS: Geriatric Depression Scale
JAGES: Japan Gerontological Evaluation Study
OR: odds ratio
PAF: population-attributable fraction
